# Implications of Lifestyle Factors and Polygenic Risk Score for Absolute Risk Prediction of Colorectal Neoplasm and Risk-Adapted Screening

**DOI:** 10.3389/fmolb.2021.685410

**Published:** 2021-07-16

**Authors:** Hongda Chen, Li Liu, Ming Lu, Yuhan Zhang, Bin Lu, Ying Zhu, Jianbo Tian, Xinying Li, Shaofa Nie, Xiaoping Miao, Min Dai

**Affiliations:** ^1^ Office of Cancer Screening, National Cancer Center/National Clinical Research Center for Cancer/Cancer Hospital, Chinese Academy of Medical Sciences and Peking Union Medical College, Beijing, China; ^2^ Department of Epidemiology and Biostatistics, and the Ministry of Education Key Lab of Environment and Health, School of Public Health, Tongji Medical College, Huazhong University of Science and Technology, Wuhan, China

**Keywords:** colorectal cancer, adenoma, polygenic risk score, risk stratification, personalized screening

## Abstract

**Background:** Estimation of absolute risk of developing colorectal neoplasm is essential for personalized colorectal cancer (CRC) screening. We developed models to determine relative and absolute risks of colorectal neoplasm based on lifestyle and genetic variants and to validate their application in risk-adapted screening.

**Methods:** We prospectively collected data from 203 advanced neoplasms, 464 non-advanced adenomas, and 1,213 healthy controls from a CRC screening trial in China in 2018–2019. The risk prediction model based on four lifestyle factors and a polygenic risk score (PRS) consisted of 19 CRC-associated single-nucleotide polymorphisms. We assessed the relative and 10-year absolute risks of developing colorectal neoplasm and the yield of a risk-adapted screening approach incorporating risk models, fecal immunochemical test, and colonoscopy.

**Results:** Compared to the participants with favorable lifestyle and lower PRS, those with unfavorable lifestyle and higher PRS had 2.87- and 3.79-fold higher risk of colorectal neoplasm in males and females, respectively. For a 50-year-old man or a 50-year-old woman with the highest risk profile, the estimated 10-year absolute risk of developing colorectal neoplasm was 6.59% (95% CI: 6.53–6.65%) and 4.19% (95% CI: 4.11–4.28%), respectively, compared to 2.80% (95% CI: 2.78–2.81%) for men and 2.24% (95% CI: 2.21–2.27%) for women with the lowest risk profile. The positive predictive value for advanced neoplasm was 31.7%, and the number of colonoscopies needed to detect one advanced neoplasm was 3.2.

**Conclusion:** The risk models, absolute risk estimates, and risk-adapted screening presented in our study would contribute to developing effective personalized CRC prevention and screening strategies.

## Introduction

Colorectal cancer (CRC) is the third most commonly diagnosed cancer and fourth leading cause of cancer-related death worldwide (Bray et al., 2018). The majority of sporadic CRC arises from normal intestinal epithelium through sequentially worsening degrees of adenomatous dysplasia (Morson, 1974; Vogelstein et al., 1988). Early detection of CRC and its precancerous lesions by means of screening has been demonstrated to be effective in reducing the mortality, even for incidence (Brenner et al., 2014; Wolf et al., 2018). To maximize the cost-effectiveness of population-based CRC screening, research studies resolving the identification of individuals at high risk and implementation of appropriate screening modalities based on risk stratification are highly valuable (Robertson and Ladabaum, 2019).

Approximately 12–35% of CRC can be attributed to genetic predisposition (Dekker et al., 2019). So far, whole-genome–wide association studies (GWASs) have identified more than 100 common genetic variants associated with the risk of CRC (Peters et al., 2013; Zhang et al., 2014; Schmit et al., 2019), among which 24 loci were validated in eastern Asian populations (Lu et al., 2019). Although individual SNPs presented modest associations with CRC, the combination of these genetic polymorphisms, known as the polygenic risk score (PRS), presented a more predominant role in CRC risk prediction (Frampton et al., 2016; Carr et al., 2020; Kastrinos et al., 2020; Thomas et al., 2020). In addition to genetic predisposition, there are several well-established modifiable lifestyle factors related to CRC (Dekker et al., 2019), including physical activity, intake of red/processed meat and dietary fruits and vegetables, lower body mass index/waist circumference, smoking, and alcohol consumption. Adherence to healthy lifestyle has been demonstrated to reduce CRC risk (Turati et al., 2017; Petimar et al., 2019; Solans et al., 2020).

Although previous studies have implied that accumulation of predisposed risk alleles and violation of healthy lifestyle are associated with increased risk of colorectal cancer (Jeon et al., 2018; Carr et al., 2020), the detailed effects of genetic predisposition and environmental exposure on colorectal adenomatous dysplasia are not completely elucidated. Moreover, the absolute risk of developing colorectal neoplasia given a specific combination of risk factors has been barely explored. In addition, from the view of translational relevance, targeting high-risk population and implementation of appropriate risk-adapted screening intervention may improve the low participation and suboptimal screening effectiveness and cost-effectiveness in population-based CRC screening programs.

Therefore, using prospectively collected samples from a large-scale population-based CRC screening trial conducted in China from 2018 through 2019, the current study was designed to estimate the relative and absolute risks of colorectal neoplasia based on the lifestyle score and the PRS and to subsequently develop and evaluate the yield of risk-adapted screening approaches incorporating risk assessment with established screening modalities including the fecal immunochemical test (FIT) and colonoscopy in detecting colorectal neoplasms.

## Materials and Methods

### Study Design and Study Sample

The study was conducted in the context of the TARGET-C trial, an ongoing study comparing the effectiveness of colonoscopy, FIT, and risk-adapted screening in CRC conducted in six centers of China since May 2018. Detailed study design has been described in previous publications (Chen et al., 2019; Chen et al., 2020). Briefly, 19,582 eligible participants aged 50–74 years were randomized into three arms in a 1:2:2 ratio: 1) one-time colonoscopy; 2) annual FIT; and 3) annual risk-adapted screening. The risk-adapted screening approach used an established CRC risk scoring system, the Asia-Pacific Colorectal Screening (APCS) score (Yeoh et al., 2011), in which participants at high risk of CRC were referred for colonoscopy, while participants at low risk were referred for FIT. All participants were required to undertake an epidemiological questionnaire survey to collect information including sociodemographic factors, history of diseases and clinical treatment, living habits, and family history of cancer. In addition, participants who needed colonoscopy examination were further required to donate stool and blood samples per standardized procedures. This study was approved by the Ethics Committee of the National Cancer Center/Cancer Hospital, Chinese Academy of Medical Sciences and Peking Union Medical College (18-013/1,615), and the protocol was registered in the Chinese Clinical Trial Registry (ChiCTR1800015506). All participants provided written informed consent.

**FIGURE 1:**
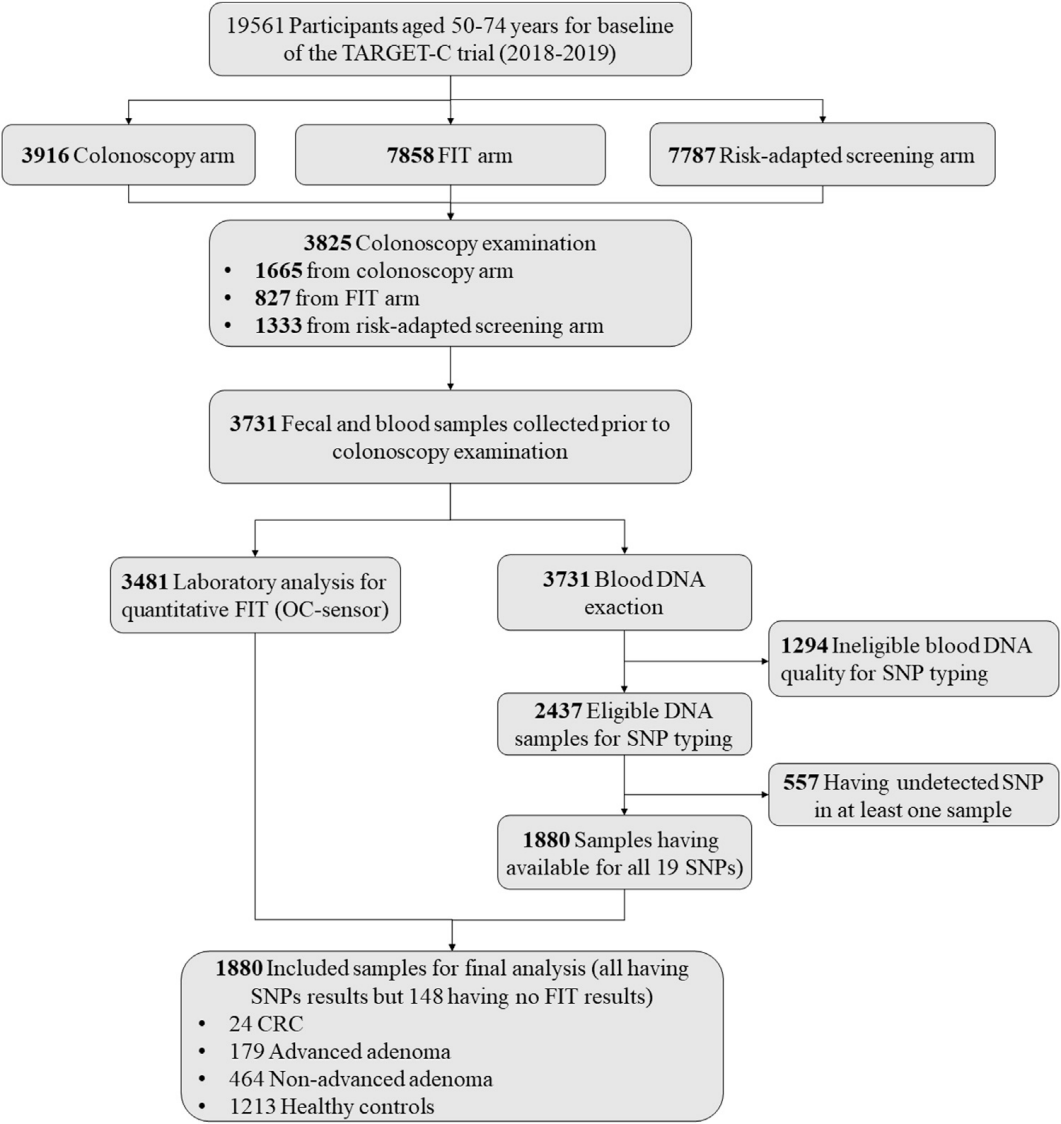
Sample selection scheme for the present study.

For the present study, we used the data and samples collected from the baseline screening phase of the TARGET-C trial. Overall, there were a total of 3,825 participants undertaking colonoscopy examination in the baseline screening phase. After excluding participants without blood samples (n = 94), having ineligible blood DNA quality for SNP typing (n = 1,294), and having failed SNP detection in at least one sample (n = 557), we finally included 1,880 samples for the final analysis, including 24 CRCs, 179 advanced adenomas, 464 non-advanced adenomas, and 1,213 controls without any significant finding at colonoscopy. The detailed sample selection scheme is presented in Figure 1.

### Blood Sample Handing and SNP Typing

Blood samples were collected in a BD Vacutainer K2 EDTA tube (no. 367863) prior to bowel preparation for colonoscopy. After collection, the samples were handled in the laboratory of the hospital within 4 h after withdrawal. The blood samples were centrifuged at 1,200 g for 12 min at room temperature, aliquoted, and stored at −80°C until further use. For the present study, DNA was extracted from buffy coat using a commercial DNA extraction kit (Tiangen, Beijing, China) according to the manufacturer’s instructions. Based on the findings from a large-scale GWAS of colorectal cancer in eastern Asia and a GWAS of advanced colorectal adenoma in Europe (Wang et al., 2013; Lu et al., 2019), 24 SNPs were initially selected and genotyped using the Sequenom MassARRAY platform, as shown in Supplementary Table S1.

### Stool Sample Handling and FIT

Eligible participants were asked to collect one stool specimen into a sterile container (SARSTEDT, Germany) from a single bowel movement, without any specific restrictions on diet or medicine within 24 h before colonoscopy examination. After collection, participants were asked to wrap the stool-filled container with an ice bag and to store it in a refrigerator at 4°C at home until their colonoscopy appointment. After receipt of the stool-filled containers, the samples were preserved at −20°C temporarily and were sent to the central biobank by cold chain logistics within one month, where the samples would be kept at −80°C for further analysis.

For the present study, the frozen fecal samples were tested by the quantitative FIT (OC-Sensor, Eiken Chemical, Japan) following a standard operating procedure in the central laboratory of the National Cancer Center, China. The laboratory staff was blinded to the colonoscopy results. Test values ≥100 ng Hb/ml (equivalent to 20 ug Hb/g, recommended cutoff by the manufacturer) were defined as positive in the present study.

### Outcome Ascertainment and Definition

Colonoscopy examinations were performed in the designated hospital by experienced endoscopists. Standardized forms recording the colonoscopy and pathology reports were collected and verified. Moreover, to ensure the uniform standard of pathology diagnosis in different study sites, an experienced gastrointestinal pathologist from the National Cancer Center, China, independently reviewed the pathology sections of all CRCs, all advanced adenomas, and a random selection of 10% of the non-advanced adenomas. For the present study, we defined advanced adenoma as having at least one of the following features: 1) high-grade dysplasia; 2) villous or tubular–villous histologic features; and 3) adenoma of 1 cm or more in diameter.

### Statistical Analysis

The distribution of demographic and lifestyle characteristics of the study population according to the screening outcomes (healthy control, non-advanced adenoma, and advanced neoplasm) was presented and compared by using the ANOVA test or chi-squared test where appropriate. Univariate logistic regression was applied to explore the associations between individual lifestyle factors and colorectal neoplasia risk stratified by sex. The lifestyle factors potentially related to colorectal neoplasia (*p* < 0.1) were selected to construct sex-specific healthy lifestyle scores, which were created by dichotomizing the information of four lifestyle factors (waist circumference, red meat intake, fruit consumption, and smoking) for men and three lifestyle factors (waist circumference, red meat intake, and fruit consumption) for women (Supplementary Table S2). Then, the weighted lifestyle score (LS) was calculated as follows: LS = β_1_χ_1_ + β_2_χ_2_ … + β_k_χ_k_… + β_n_χ_n_, where β_k_ is the log-odds ratio (OR) for colorectal neoplasm related to the lifestyle factor k and χ_k_ is the value of the lifestyle factor k. After exclusion of five SNPs with statistical departure from the Hardy–Weinberg equilibrium (HWE), 19 SNPs were used to construct the PRS. The details of the SNPs are listed in Supplementary Table S1. Each SNP was coded as 0, 1, or 2 copies of the risk allele. The weighted PRS was calculated as follows: PRS = β_1_χ_1_ + β_2_χ_2_ … + β_k_χ_k_ … + β_n_χ_n_, where β_k_ is the per-allele log-OR for colorectal neoplasm related to SNP k and χ_k_ is the allele dosage of SNP k. The weights for each SNP included in the PRS are presented in Supplementary Table S3.

Multiple logistic regression was used to estimate the associations of the healthy lifestyle score, the PRS, and the combination of these two scores with the risk of colorectal neoplasm, non-advanced colorectal adenoma, and advanced colorectal neoplasm by calculating ORs and 95% confidence intervals (CIs). We stratified the model by sex to allow for potential difference in the associations between men and women. In these analyses, the lifestyle score was dichotomized as unfavorable and favorable according to the sex-specific median of the score, with higher scores indicating unfavorable and lower scores representing favorable. The weighted PRS was categorized into two groups according to the 90th percentile of its distribution (higher PRS: ≥ the 90th percentile; lower PRS: < the 90th percentile). The subgroup analysis was conducted according to age and sex of the participants.

We further estimated the 10-year absolute risk and its 95% CIs for developing colorectal neoplasm for 50-year-old men and women with specific profiles of lifestyle score and PRS, based on the principles of modeling described by Freedman et al. (2009), Pfeiffer et al. (2011). Briefly, the estimation of the absolute risk of colorectal neoplasm includes estimating relative risk of colorectal neoplasm (calculated from a population-based case–control study) and attributable risk parameters and combining these estimates with baseline age-specific cancer hazard rates from the Chinese Center for Cancer Registry Data to estimate the probability of developing colorectal neoplasm during a specific time interval given a person’s age, lifestyle score, and PRS. As colorectal neoplasm tends to occur at old ages, we accounted for the competing risks of non-CRC–specific mortality in the absolute risk estimation. The details of the calculation have been provided by Carr et al. (2020).

**FIGURE 2:**
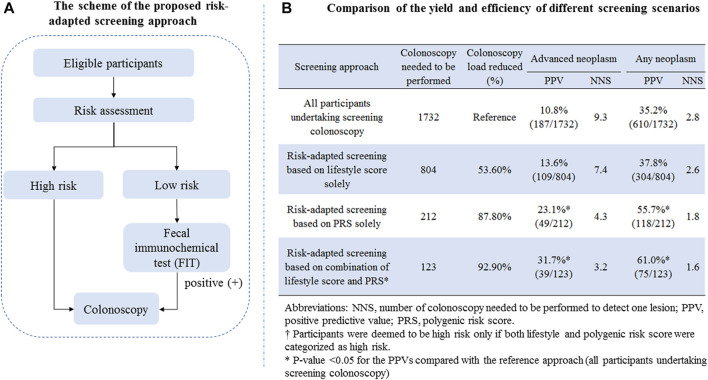
**(A)** Scheme of the proposed risk-adapted screening approach; **(B)** comparison of the yield and efficiency of different screening scenarios.

Based on risk stratification using environmental exposure and PRS described above, we designed a risk-adapted screening approach as shown in Figure 2A. Briefly, all subjects firstly involved in risk assessment; for subjects assessed to be at high risk, colonoscopy was recommended; for subjects assessed to be at low risk, FIT was offered; and those with positive test results were further offered colonoscopy. To determine the yield of efficiency of the risk-adapted screening scenarios and their comparison with the traditional colonoscopy-only approach, we calculated the positive predictive values (PPVs) for detecting advanced neoplasm (including CRC or advanced adenoma) and any neoplasm (including CRC, advanced adenoma, and non-advanced adenoma). The PPV was defined as the number of patients with true diagnosis of interest divided by the number of subjects deemed positive. To assess the resource load of colonoscopy, we calculated the number of colonoscopies needed to be screened (NNS) to detect one lesion.

All analyses were performed using SAS (version 9.4, SAS Institute Inc., Cary, NC) and R software (version 3.5.1) (ore Team (2018). R: A, 2018), and all statistic tests were two-sided, with the *p* value less than 0.05 being statistically significant.

## Results

### Basic Characteristics

A detailed sample selection scheme from the TARGET-C study is shown in Figure 1. Overall, 1880 participants were finally included into analysis, consisting of 203 advanced neoplasia cases (24 CRCs and 179 advanced adenomas), 464 non-advanced adenomas, and 1,213 healthy controls (Table 1). The mean age of the participants was 60.5 years, and 51.3% were male. Compared with healthy controls, colorectal neoplasm cases, including non-advanced adenoma and advanced neoplasm, were more likely to be male, older, and obese and to have more consumption of red meat but less intake of fruits. In addition, colorectal neoplasm cases had a higher lifestyle score and PRS compared with health controls (Table 1).

**TABLE 1:** Basic characteristics of included participants.

Characteristics	Total (n = 1880)	Healthy controls (n = 1,213)	Non-advanced adenoma (n = 464)	Advanced neoplasm (n = 203)	*p* value^a^
Sex, n (%)					<0.0001
Female	916 (48.7)	700 (57.7)	158 (34.1)	58 (28.6)	
Male	964 (51.3)	513 (42.3)	306 66.0)	145 (71.4)	
Age, mean (standard deviation), years	60.5 (6.3)	59.8 (6.3)	61.5 (6.1)	61.7 (6.3)	<0.0001
Smoking status, n (%)					
Male, ≥15 pack-years	464 (48.1)	228 (44.4)	159 (52.0)	77 (53.1)	0.05
Female, >0 pack-years	14 (1.5)	9 (1.3)	4 (2.5)	1 (1.7)	0.51
Alcohol consumption (> 250 ml alcohol/week), n (%)					
Male	181 (18.8)	91 (17.7)	54 (17.7)	36 (24.8)	0.13
Female	17 (1.9)	14 (2.0)	2 (1.3)	1 (1.7)	0.82
Waistline, mean (standard deviation), cm					
Male	86.9 (11.1)	86.1 (11.3)	87.2 (11.2)	89.5 (9.8)	0.004
Female	81.4 (11.3)	80.7 (11.5)	83.0 (11.0)	84.9 (8.8)	0.004
Red meat consumption, n (%)					
Male	901 (93.5)	475 (92.6)	285 (93.1)	141 (97.2)	0.13
Female	798 (91.4)	602 (86.0)	143 (90.5)	53 (91.4)	0.19
Fruit consumption, n (%)					
Male	585 (60.7)	298 (58.1)	196 (64.1)	91 (62.8)	0.21
Female	477 (52.1)	348 (49.7)	91 (57.6)	38 (65.5)	0.02
Physically active^b^, n (%)					
Male	421 (43.7)	237 (46.2)	124 (40.5)	60 (41.4)	0.24
Female	386 (42.1)	299 (42.7)	64 (40.5)	23 (39.7)	0.81
Polygenic risk score, median (interquartile range) Lifestyle score, median (interquartile range)	0.25 (0.05–0.46)	0.22 (0.02–0.42)	0.32 (0.12–0.53)	0.27 (0.08–0.53)	<0.0001
Male	0.74 (0.49–0.92)	0.68 (0.49–0.92)	0.81 (0.49–0.92)	0.81 (0.58–1.00)	<0.0001
Female	0.80 (0.47–0.87)	0.78 (0.47–0.87)	0.87 (0.47–1.20)	0.87 (0.80–1.20)	0.0003

Advanced neoplasm included colorectal cancer and advanced adenoma. Favorable controls indicated no significant findings at colonoscopy examination.

a *p* values were calculated by conducting the chi-square test for categorical variables or ANOVA test for continuous variables.

b Physically active was defined as at least 1 h moderate-to-intense leisure time physical activity per week.

### Association of Lifestyle Score and PRS With Colorectal Neoplasm Risk

Unfavorable lifestyle was associated with increased risk of any colorectal neoplasm, non-advanced adenoma, and advanced neoplasm, with the ORs of 1.68 (95% CI: 1.30–2.17), 1.53 (95% CI: 1.15–2.03), and 2.04 (95% CI: 1.40–3.00) for males and 1.85 (95% CI: 1.32–2.58), 1.72 (95% CI: 1.18–2.51), and 2.26 (95% CI: 1.22–4.21) for females. Higher PRSs presented positive association with the risk of colorectal neoplasm, non-advanced adenoma, and advanced neoplasm, with the ORs of 1.83 (95% CI: 1.21–2.75), 1.79 (95% CI: 1.14–2.80), and 1.87 (95% CI: 1.07–3.27) for males and 2.08 (95% CI: 1.27–3.40), 2.40 (95% CI: 1.41–4.07), and 1.30 (95% CI: 0.49–3.42) for females (Supplementary Table S4). Similar risk effects of unfavorable lifestyle and higher PRS on colorectal neoplasm, non-advanced adenoma, and advanced neoplasm have been found in the sensitivity analysis by adding a lifestyle–PRS interaction item in logistic regression models, although the 95% confidence intervals of the ORs became much wider due to limited samples of women (Supplementary Table S5). The comparisons of the risk of colorectal neoplasm, non-advanced adenoma, and advanced neoplasm with the lifestyle score and genetic score according to age increase are presented in Supplementary Figure S1. The combined effects of lifestyle score and PRS on colorectal neoplasia were further detected. Compared to the participants with favorable lifestyle and lower PRS, those with unfavorable lifestyle and higher PRS had 2.87-, 2.41-, and 3.96-fold risk of colorectal neoplasm, non-advanced adenoma, and advanced neoplasm in males and 3.79-, 4.26-, and 2.50-fold risk of colorectal neoplasm, non-advanced adenoma, and advanced neoplasm in females (Table 2). Further analyses stratified by age revealed the adverse effect of unfavorable lifestyle and higher PRS was more apparent in younger age in males, but in older age in females (Supplementary Table S6). In the sensitivity analysis where the PRSs were categorized into the higher genetic risk group (Tertile 3) and lower genetic risk group (Tertiles 1 and 2), similar but attenuated associations between the risk profiles and colorectal neoplasm were observed (Supplementary Table S7).

**TABLE 2:** Odds ratios of risk profiles with colorectal neoplasm, non-advanced adenoma, and advanced neoplasm stratified by sex.

	Any neoplasm		Non-advanced adenoma		Advanced neoplasm	
	No. of cases/no. of controls	OR (95% CI)	No. of cases/no. of controls	OR (95% CI)	No. of cases/no. of controls	OR (95% CI)
**Male**						
Favorable lifestyle and lower PRS	166/264	1	118/264	1	48/264	1
Favorable lifestyle and higher PRS	28/22	2.01 (1.11–3.64)	21/22	2.12 (1.12–4.02)	7/22	1.73 (0.70–4.28)
Unfavorable lifestyle and lower PRS	219/205	1.71 (1.30–2.25)	144/205	1.59 (1.17–2.15)	75/205	2.01 (1.34–3.02)
Unfavorable lifestyle and higher PRS	38/22	2.87 (1.64–5.04)	23/22	2.41 (1.29–4.51)	15/22	3.96 (1.91–8.22)
**Female**						
Favorable lifestyle and lower PRS	52/270	1	40/270	1	12/270	1
Favorable lifestyle and higher PRS	9/22	2.14 (0.93–4.92)	7/22	2.18 (0.87–5.45)	2/22	2.05 (0.43–9.76)
Unfavorable lifestyle and lower PRS	135/381	1.86 (1.30–2.65)	94/381	1.69 (1.13–2.53)	41/381	2.43 (1.25–4.71)
Unfavorable lifestyle and higher PRS	20/27	3.79 (1.98–7.28)	17/27	4.26 (2.13–8.53)	3/27	2.50 (0.67–9.42)

PRS: polygenic risk score; OR: odds ratio; CI: confidence interval.

Lifestyle was binarized as favorable and unfavorable according to the median of the lifestyle score.

Genetic risk was categorized as lower and higher according to the 90th percentile of the polygenic risk score.

### Absolute Risk Estimates for Colorectal Neoplasm Based on Lifestyle Score and PRS

The attributable risk estimates for colorectal neoplasm stratified by age and sex are presented in Supplementary Table S8. The estimated colorectal adenoma incidence in China is listed in Supplementary Table S9. Based on the national death registry data and cancer registry information, we summarized the mortality for men and women in China in 2015 in Supplementary Table S10. Table 3 presents the estimates of the 10-year absolute risk of developing colorectal neoplasm for males and females separately, aged 50 years, combining information on the lifestyle score and PRS. The 10-year absolute risk of developing colorectal neoplasm varied across risk profiles in males and females. To illustrate, for a 50-year-old man with the highest risk profile (unfavorable lifestyle and higher PRS), the estimated 10-year absolute risk of developing colorectal neoplasm was 6.59% (95% CI: 6.53–6.65%), compared to 2.80% (95% CI: 2.78–2.81%) for men with the lowest risk profile (adherence to favorable lifestyle and lower PRS). For a 50-year-old woman with the highest risk profile, the estimated 10-year absolute risk of developing colorectal neoplasm was 4.19% (95% CI: 4.11–4.28%), compared to 2.24% (95% CI: 2.21–2.27%) for females with favorable lifestyle and lower PRS (Table 3).

**TABLE 3:** 10-year absolute risk estimates of colorectal neoplasm for 50-year-old men and women.

Subgroup	10-year absolute risk (95% CI)	
	Men	Women
Favorable lifestyle		
Lower PRS	2.80 (2.78–2.81)	2.24 (2.21–2.27)
Higher PRS	6.12 (6.04–6.20)	4.08 (4.00–4.16)
Unfavorable lifestyle		
Lower PRS	4.56 (4.54–4.58)	3.60 (3.58–3.62)
Higher PRS	6.59 (6.53–6.65)	4.19 (4.11–4.28)

Abbreviations: CI, confidence interval: PRS, polygenic risk score.

Lifestyle was binarized as favorable and unfavorable according to the median of the lifestyle score.

Genetic risk was categorized as lower and higher according to the 90th percentile of the polygenic risk score.

### Yield of Risk-Adapted Screening

To evaluate the translation potential of the constructed risk assessment score in CRC screening, we proposed a risk-adapted screening approach incorporating risk assessment, FIT, and colonoscopy, and the detailed scheme is shown in Figure 2A. The detailed comparison of the yield and resource load of different screening scenarios is shown in Figure 2B. For the traditional approach in that all participants aged 50–74 years undertake screening colonoscopy, the PPV and NNS for detecting advanced neoplasm were 10.8% and 9.3, respectively; those for detecting any neoplasm were 35.2% and 2.8, respectively. Setting the yield of the colonoscopy-only screening approach as a reference, the risk-adapted screening approach showed remarkably increased PPVs, decreased colonoscopy load, and lower NNSs for detecting colorectal neoplasm. The strongest improved PPV and NNS were observed for risk-adapted screening combining the lifestyle score and PRS, yielding the PPV and NNS of 31.7% and 3.2 for detecting advanced neoplasm, respectively, and 61.0% and 1.6 for detecting any neoplasm.

## Discussion

Using samples from the population-based CRC screening trial conducted in China, we presented the relative risk and 10-year absolute risk estimates for developing colorectal neoplasm considering the lifestyle score and PRS. Moreover, we further empirically demonstrated that the risk-adapted CRC screening incorporating risk assessment and established screening modalities has great translational potential in terms of increasing PPV for detecting advanced neoplasm and reducing resource load of colonoscopy. Such findings of our study may therefore have strong implications in future CRC screening, which may aid the suboptimal participation and efficiency of the current one-size-fits-all screening strategies, especially for countries with relatively low disease burden and limited healthcare resources.

The relative risk estimates of the genetic or lifestyle factors with colorectal neoplasm have been reported previously (Wang et al., 2013; Bailie et al., 2017). To our knowledge, this is the first estimate of absolute risk for developing colorectal neoplasm based on genetic and lifestyle factors. The absolute risk results support our hypothesis that lifestyle factors may modify the risk of colorectal neoplasm. The 10-year absolute risk associated with unfavorable lifestyle was greatest in the group at higher genetic risk, which also emphasizes the benefit of adhering to healthy lifestyle. Within the lower polygenic risk category, unfavorable lifestyle resulted in about 60% higher 10-year absolute risk of colorectal neoplasm in both males and females when compared to favorable lifestyle, suggesting that the genetic insusceptibility of colorectal neoplasm can be offset by an unhealthy lifestyle. In addition, although one may perceive that having a higher genetic risk score means powerless against genetic predisposition, the current study implies that a healthy lifestyle and a personalized CRC screening, at least to some extent, may reduce the risk of colorectal neoplasm (Carr et al., 2020). The current study added more values of genetic and lifestyle factors in the risk prediction of colorectal precancerous lesions, which provides a validation for previous findings of the health effects of genetic and lifestyle factors on colorectal carcinogenesis.

The estimate of absolute risk of CRC has implications of personalized CRC screening. Previously, Joen J and colleagues (Jeon et al., 2018) derived the risk prediction models using 9 lifestyle and environmental factors and 63 SNPs, and the results showed that the starting ages of screening varied significantly for individuals with different risk profiles, although screening is recommended to begin at 50 years for individuals with no family history of CRC. In addition, Frampton M. J. and colleagues (Frampton et al., 2016) derived a PRS using 37 SNPs and calculated absolute risk of CRC from the United Kingdom population age structure, and they found that personalized screening using the PRS would then result in 26% fewer men and women being eligible for screening with 7 and 5% fewer screen-detected cases. For our study, due to lack of evidence of reference absolute risk of colorectal adenoma, we therefore did not provide recommendation of personalized starting age based on the risk profiles. However, the absolute risk estimates can still be useful to facilitate communication and to better inform the public about the magnitude and potentials of CRC prevention and may help to define those likely to maximally benefit from chemoprevention and screening.

In our previous study (Chen et al., 2020), we demonstrated that the risk-adapted screening approach incorporating the environmental risk assessment, FIT, and colonoscopy had satisfying participation rate and superior yield than the FIT-based screening strategy in population-based CRC screening. The current study finding expanded our previous research and indicated that the risk-adapted screening approach incorporating the PRS additionally may further improve the screening yield in terms of higher PPVs for advanced neoplasm and lower NNSs. However, such validation was only validated in a retrospective manner, and technical issues and operational details such as optimal positivity threshold of risk score, population compliance rate, and health resource allocation need to be considered carefully before such a strategy is introduced. In addition, with the development of the novel early detection biomarkers, how to incorporate the PRS with established and novel screening modalities deserves further research.

In the present study, the SNPs used for the PRS were selected based on previous GWAS findings, especially for eastern Asian population. There were several issues needed to be taken into consideration when comparing our results with others. First, the discovered SNPs associated with advanced adenoma are rather limited; therefore, the prediction accuracy of the constructed PRS may not be optimal given the majority of the samples were actually adenomas from a CRC screening setting. Second, there were some disparities regarding the CRC-associated SNPs between the Asian and European populations. For our study, we specifically selected some validated CRC-associated SNPs in the eastern Asian populations, which may not be the susceptibility genes in the other populations. Third, we adopted a targeted genotyping approach using the Sequenom MassARRAY platform rather than using the whole-genome sequencing, which only detected a limited number of SNPs simultaneously. We anticipated that the risk prediction accuracy could be further enhanced, if more colorectal neoplasm–associated SNPs were included in the PRS. However, from the view of translational use in population-based screening, the proposed PRS should have the merit of affordable cost and convenient-to-detect nature.

Our study has specific strengths**.** To our knowledge, this is the first study to estimate the absolute risk of colorectal neoplasm based on genetic and lifestyle factors in Chinese population. The current study estimates the probability of developing colorectal neoplasm over a 10-year time interval using data from a nation-wide colonoscopy screening program and a multicentered CRC screening trial. Thus, it is expected that the risk prediction is to some extent representative of the general China population. Moreover, the lifestyle and genetic factors involved in the prediction model can be attained in the clinical setting, which provides more practical meaning for generalization.

Our study also has some limitations. First, the sample size was relatively small, which disenabled the combination of lifestyle and genetic information in a very detailed way. Second, all lifestyle factors were collected retrospectively by interview; therefore, recall bias might not be avoided. However, since all information was collected prior to colonoscopy, this recall bias might cause undeferential misclassification of exposures, which consequently resulted in an underestimated effect of unhealthy lifestyle. Third, the numbers of included SNPs in the PRS were limited.

To sum up, using samples from a multicentered CRC screening trial conducted in China, we presented the relative risk and 10-year absolute risk estimates for developing colorectal neoplasm considering the lifestyle score and genetic predisposition. Retrospective analysis revealed that the proposed risk-adapted screening approach incorporating the lifestyle score, PRS, FIT, and colonoscopy showed improved screening efficiency than the traditional colonoscopy-based screening approach. The proposed risk prediction models and the risk-adapted screening approach would contribute to the development of effective personalized CRC prevention and screening strategies in the future.

## Data Availability

The raw data supporting the conclusion of this article will be made available upon reasonable request to the corresponding author.
