# Supplementary material for: Implications of Lifestyle Factors and Polygenic Risk Score for Absolute Risk Prediction of Colorectal Neoplasm and Risk-Adapted Screening
Source: Front Mol Biosci. 2021 Jul 16;8:685410. doi: 10.3389/fmolb.2021.685410 (PMC8324207; doi:10.3389/fmolb.2021.685410)
Supplement: Supplementary file 1 [file DataSheet1.docx]

**Online Supplementary Materials**

**Table S1.** Details of the selected SNPs for genotyping

**Table S2.** Description of the lifestyle factors used to derive the lifestyle score

**Table S3.** The weights of each SNP used to construct the polygenic risk score

**Table S4.** Odds ratios of lifestyle score and polygenic risk score associated with colorectal neoplasm, non-advanced adenoma and advanced neoplasm stratified by sex

**Table S5.** Sensitivity analysis for the odds ratios of lifestyle score and polygenic risk score associated with colorectal neoplasm, non-advanced adenoma and advanced neoplasm stratified by sex

**Table S6.** Odds ratios of risk profiles associated with colorectal neoplasm stratified by sex and age

**Table S7.** Sensitivity analysis of the odds ratios of risk profiles associated with colorectal neoplasm stratified by sex

**Table S8.** Attributable risk estimates for colorectal neoplasm stratified by sex and age

**Table S9.** The estimated colorectal adenoma incidence (per 100,000) in China in 2015

**Table S10.** The mortality (deaths per 100,000) for men and women in China in 2015

**Figure S1**. Odds ratios of E-score (lifestyle score) and G-score (polygenic risk score) according to sex and age. (A) ORs of lifestyle score for colorectal neoplasm; (B) ORs of lifestyle score for non-advanced adenoma; (C). ORs of lifestyle score for advanced adenoma; (D) ORs of PRS for colorectal neoplasm; (E) ORs of PRS for non-advanced adenoma; (F). ORs of PRS for advanced adenoma.

Table S1. Details of the selected SNPs for genotyping

| rsID | Chr | Gene | Call rate | *P*_HWE_ |
| --- | --- | --- | --- | --- |
| rs7542665 | 1p31.3 | missense of L1TD1 | 0.98 | 0.720501 |
| rs7606562 | 2p16.3 | intronic of PPP1R21 | 0.98 | 0.786354 |
| rs17651822 | 3p24.1 | LINC00693 | 0.96 | 0.151364 |
| **rs113569514** | **3q22.2** | **5'UTR of SLCO2A1** | **0.98** | **0.041604** |
| rs12659017 | 5q23.2 | intergenic (ALDH7A1, PHAX ) | 0.98 | 0.885603 |
| rs647161 | 5q31.1 | PITX1 | 0.98 | 0.450943 |
| rs3830041 | 6p21.32 | intronic of NOTCH4 | 0.99 | 0.170436 |
| rs1476570 | 6p22.1 | intronic of HLA-G | 0.98 | 0.687535 |
| rs2450115 | 8q23.3 | EIF3H | 0.98 | 0.953775 |
| rs6469656 | 8q23.3 | EIF3H | 0.99 | 0.520772 |
| rs6983267 | 8q24.21 |  | 0.98 | 0.641653 |
| rs704017 | 10q22.3 | ZMIZ1 | 0.98 | 0.45175 |
| rs6584283 | 10q24.2 | 2kb 5' of NKX2-3 | 0.97 | 0.426809 |
| rs11196172 | 10q25.2 | TCF7L2 | 0.98 | 0.949202 |
| rs12241008 | 10q25.2 | VTI1A | 0.98 | 0.830436 |
| **rs174537** | **11q12.2** | **MYRF** | **0.97** | **0.015415** |
| rs2730985 | 12q12 | 147kb 5' of PRICKLE1 | 0.98 | 0.95219 |
| **rs10774214** | **12p13.32** | **CCND2** | **0.34** | **1.53E-89** |
| rs1886450 | 13q22.1 | intergenic (KLF5, KLF12) | 0.98 | 0.954649 |
| rs4341754 | 16q23.2 | intergenic (WWOX, MAF) | 0.98 | 0.865501 |
| **rs1078643** | **17p12** | **19kb 3' of PIRT** | **0.98** | **0.039155** |
| rs6086235 | 20p12.3 | 53kb 3' of HAO1 | 0.98 | 0.340248 |
| rs8121252 | 20p13.32 | GNAS intronic | 0.98 | 0.506796 |
| **rs2837254** | **21q22.2** |  | **0.98** | **0.005898** |

rs113569514, rs174537, rs10774214, rs1078643 and rs2837254 were excluded from polygenic risk score construction due to departure from Hardy Weinberg Equilibrium (HWE).

Table S2. Description of the lifestyle factors used to derive the lifestyle score

| Lifestyle factor | Point | Description | Weights of each lifestyle factor when constructing the lifestyle score* |
| --- | --- | --- | --- |
| Waist | 0 | Waist ≤ 82 cm for women or waist ≤ 88 cm for men |  |
|  | 1 | Waist > 82 cm for women or Waist > 88 cm for men | 0.3280 for women; 0.4227 for men |
| Red meat intake | 0 | ≤1 serving/week for women; ≤1 serving/day for men; |  |
|  | 1 | >1 serving/week for women; >1 serving/day for men | 0.4671 for women; 0.2607 for men |
| Fruit intake | 0 | ≥1 serving/day |  |
|  | 1 | <1 serving/day | 0.4053 for women; 0.2332 for men |
| Smoking (men only) | 0 | <15 pack-years |  |
|  | 1 | ≥15 pack-years | 0.3163 for men |

*The weight of each lifestyle factor was the log-odds ratio (OR) for colorectal neoplasm related to that factor.

**Table S3.** The weights of each SNP used to construct the polygenic risk score

| SNP | Weights |
| --- | --- |
| rs11196172 | -0.2336 |
| rs12241008 | 0.0993 |
| rs12659017 | -0.1277 |
| rs1476570 | 0.1468 |
| rs17651822 | -0.109 |
| rs1886450 | 0.0117 |
| rs2450115 | -0.0769 |
| rs2730985 | 0.0265 |
| rs3830041 | -0.0517 |
| rs4341754 | -0.0626 |
| rs6086235 | 0.0828 |
| rs6469656 | 0.0592 |
| rs647161 | 0.1495 |
| rs6584283 | 0.041 |
| rs6983267 | 0.1221 |
| rs704017 | -0.00568 |
| rs7542665 | 0.207 |
| rs7606562 | -0.0833 |
| rs8121252 | 0.0729 |

Table S4. Odds ratios of lifestyle score and polygenic risk score associated with colorectal neoplasm, non-advanced adenoma and advanced neoplasm stratified by sex

|  | Any neoplasm | |  | Non-advanced adenoma | |  | Advanced neoplasm | |
| --- | --- | --- | --- | --- | --- | --- | --- | --- |
|  | No. Case /No. Control | OR (95% CI) |  | No. Case /No. Control | OR (95% CI) |  | No. Case /No. Control | OR (95% CI) |
| Male |  |  |  |  |  |  |  |  |
| Lifestyle |  |  |  |  |  |  |  |  |
| Favorable | 194/286 | 1 |  | 139/286 | 1 |  | 55/286 | 1 |
| Unfavorable | 257/227 | 1.68 (1.30-2.17) |  | 167/227 | 1.53 (1.15-2.03) |  | 90/227 | 2.04 (1.40-3.00) |
| Polygenic risk score |  |  |  |  |  |  |  |  |
| Lower | 385/469 | 1 |  | 262/469 | 1 |  | 123/469 | 1 |
| Higher | 66/44 | 1.83 (1.21-2.75) |  | 44/44 | 1.79 (1.14-2.80) |  | 22/44 | 1.87 (1.07-3.27) |
|  |  |  |  |  |  |  |  |  |
| Female |  |  |  |  |  |  |  |  |
| Lifestyle |  |  |  |  |  |  |  |  |
| Favorable | 61/292 | 1 |  | 47/292 | 1 |  | 14/292 | 1 |
| Unfavorable | 155/408 | 1.85 (1.32-2.58) |  | 111/408 | 1.72 (1.18-2.51) |  | 44/408 | 2.26 (1.22-4.21) |
| Polygenic risk score |  |  |  |  |  |  |  |  |
| Lower | 187/651 | 1 |  | 134/651 | 1 |  | 53/651 | 1 |
| Higher | 29/49 | 2.08 (1.27-3.40) |  | 24/49 | 2.40 (1.41-4.07) |  | 5/49 | 1.30 (0.49-3.42) |

PRS: polygenic risk score; OR: odds ratio; CI: confidence interval.

Lifestyle was binarized as favorable and unfavorable according to the median of the lifestyle score.

Genetic risk was categorized as lower and higher according to the 90^th^ percentile of the polygenic risk score.

Table S5. Sensitivity analysis for the odds ratios of lifestyle score and polygenic risk score associated with colorectal neoplasm, non-advanced adenoma and advanced neoplasm stratified by sex*

|  | Any neoplasm | |  | Non-advanced adenoma | |  | Advanced neoplasm | |
| --- | --- | --- | --- | --- | --- | --- | --- | --- |
|  | OR (95% CI) | *P*_interaction_ |  | OR (95% CI) | *P*_interaction_ |  | OR (95% CI) | *P*_interaction_ |
| Male |  |  |  |  |  |  |  |  |
| Lifestyle |  | 0.46 |  |  | 0.54 |  |  | 0.52 |
| Favorable | 1 |  |  | 1 |  |  | 1 |  |
| Unfavorable | 2.32 (0.84-6.36) |  |  | 2.07 (0.68-6.31) |  |  | 2.82 (0.72-11.12) |  |
| Polygenic risk score |  |  |  |  |  |  |  |  |
| Lower | 1 |  |  | 1 |  |  | 1 |  |
| Higher | 2.03 (1.24-3.33) |  |  | 1.95 (1.13-3.36) |  |  | 2.18 (1.10-4.32) |  |
|  |  |  |  |  |  |  |  |  |
| Female |  |  |  |  |  |  |  |  |
| Lifestyle |  | 0.93 |  |  | 0.80 |  |  | 0.50 |
| Favorable | 1 |  |  | 1 |  |  | 1 |  |
| Unfavorable | 1.95 (0.58-6.49) |  |  | 1.46 (0.39-5.48) |  |  | 4.83 (0.49-48.04) |  |
| Polygenic risk score |  |  |  |  |  |  |  |  |
| Lower | 1 |  |  | 1 |  |  | 1 |  |
| Higher | 2.14 (0.93-4.92) |  |  | 2.18 (0.87-5.45) |  |  | 2.05 (0.43-9.76) |  |

PRS: polygenic risk score; OR: odds ratio; CI: confidence interval.

Lifestyle was binarized as favorable and unfavorable according to the median of the lifestyle score.

Genetic risk was categorized as lower and higher according to the 90^th^ percentile of the polygenic risk score.

*The interaction item between lifestyle and genetic risk score was added into the logistic regression models.

Table S6. Odds ratios of risk profiles associated with colorectal neoplasm stratified by sex and age

| Subgroup | No. Case/No. Controls | |  | OR (95% CI) | |
| --- | --- | --- | --- | --- | --- |
|  | Men | Women |  | Men | Women |
| Age: 50-59 y |  |  |  |  |  |
| Favorable lifestyle & lower PRS | 44 /98 | 29/158 |  | 1.00 (Ref) | 1.00 (Ref) |
| Favorable lifestyle & higher PRS | 10/7 | 5/13 |  | 3.18 (1.14-8.91) | 2.10 (0.69-6.33) |
| Unfavorable lifestyle & lower PRS | 65/75 | 68/208 |  | 1.93 (1.19-3.14) | 1.78 (1.10-2.88) |
| Unfavorable lifestyle & higher PRS | 15/9 | 6/15 |  | 3.71 (1.51-9.13) | 2.18 (0.78-6.08) |
| Age≥60 y |  |  |  |  |  |
| Favorable lifestyle & lower PRS | 122/166 | 23/112 |  | 1.00 (Ref) | 1.00 (Ref) |
| Favorable lifestyle & higher PRS | 18/15 | 4/9 |  | 1.63 (0.79-3.37) | 2.16 (0.61-7.63) |
| Unfavorable lifestyle & lower PRS | 154/130 | 67/173 |  | 1.61 (1.16-2.24) | 1.89 (1.11-3.20) |
| Unfavorable lifestyle & higher PRS | 23/13 | 14/12 |  | 2.41 (1.17-4.94) | 5.68 (2.33-13.86) |

PRS: polygenic risk score; OR: odds ratio; CI: confidence interval.

Lifestyle was binarized as favorable and unfavorable according to the median of the lifestyle score.

Genetic risk was categorized as lower and higher according to the 90^th^ percentile of the polygenic risk score.

Table S7. Sensitivity analysis of the odds ratios of risk profiles associated with colorectal neoplasm stratified by sex

|  | Any neoplasm | |  | Non-advanced adenoma | |  | Advanced neoplasm | |
| --- | --- | --- | --- | --- | --- | --- | --- | --- |
|  | No. Case /  No. Control | OR (95% CI) |  | No. Case /  No. Control | OR (95% CI) |  | No. Case /  No. Control | OR (95% CI) |
| Male |  |  |  |  |  |  |  |  |
| Favorable lifestyle & lower PRS | 117/188 | 1 |  | 84/188 | 1 |  | 33/188 | 1 |
| Favorable lifestyle & higher PRS | 77/98 | 1.28 (0.88-1.88) |  | 55/98 | 1.27 (0.84-1.94) |  | 22/98 | 1.27 (0.71-2.33) |
| Unfavorable lifestyle & lower PRS | 136/153 | 1.45 (1.04-2.01) |  | 89/153 | 1.32 (0.91-1.91) |  | 47/153 | 1.75 (1.07-2.87) |
| Unfavorable lifestyle & higher PRS | 121/74 | 2.71 (1.87-3.93) |  | 78/74 | 2.42 (1.61-3.65) |  | 43/74 | 3.39 (2.00-5.77) |
| Female |  |  |  |  |  |  |  |  |
| Favorable lifestyle & lower PRS | 31/199 | 1 |  | 21/199 | 1 |  | 10/199 | 1 |
| Favorable lifestyle & higher PRS | 30/93 | 2.08 (1.19-3.64) |  | 26/93 | 2.69 (1.43-5.04) |  | 4/93 | 0.86 (0.26-2.81) |
| Unfavorable lifestyle & lower PRS | 90/272 | 2.15 (1.37-3.36) |  | 59/272 | 2.09 (1.23-3.57) |  | 31/272 | 2.27 (1.09-4.75) |
| Unfavorable lifestyle & higher PRS | 65/136 | 3.07 (1.90-4.97) |  | 52/136 | 3.67 (2.11-6.39) |  | 13/136 | 1.91 (0.81-4.49) |

PRS: polygenic risk score; OR: odds ratio; CI: confidence interval.

Lifestyle was binarized as favorable and unfavorable according to the median of the lifestyle score.

Genetic risk was categorized according to the tertiles of the polygenic risk score. Tertile 1 and Tertile 2 of the polygenic risk score were combined into lower PRS, and Tertile 3 was classified as higher PRS.

Table S8. Attributable risk estimates for colorectal neoplasm stratified by sex and age

| Sex, age group | N | Case patients, n | Attributable risk |
| --- | --- | --- | --- |
| Men, <60 y | 323 | 134 | 0.37 |
| Men, ≥60 y | 641 | 317 | 0.25 |
| Women, <60 y | 502 | 107 | 0.33 |
| Women, ≥60 y | 414 | 108 | 0.42 |

Table S9. The estimated colorectal adenoma incidence (per 100,000) in China in 2015

| Age group (y) | Men | Women |
| --- | --- | --- |
| 50-54 | 655 | 387 |
| 55-59 | 793 | 483 |
| 60-64 | 1047 | 681 |
| 65-69 | 1132 | 792 |

A birth cohort analysis similar to the approach proposed by Brenner H was performed to estimate the adenoma incidence in China in 2015 by using the published population-based colonoscopy screening data in China (2014-2016).

References:

1. Brenner H, et al. Natural history of colorectal adenomas: birth cohort analysis among 3.6 million participants of screening colonoscopy. Cancer Epidemiol Biomarkers Prev. 2013 Jun;22(6):1043-1051.

2. Brenner H, et al. Incidence of colorectal adenomas: birth cohort analysis among 4.3 million participants of screening colonoscopy. Cancer Epidemiol Biomarkers Prev. 2014 Sep;23(9):1920-1927)

3. Chen H, et al. Participation and yield of a population-based colorectal cancer screening programme in China. Gut. 2019 Aug;68(8):1450-1457.

Table S10. The mortality (deaths per 100,000) for men and women in China in 2015

| Age (y) | Male | | |  | Female | | |
| --- | --- | --- | --- | --- | --- | --- | --- |
|  | All causes | Colorectal cancer-specific | Other causes |  | All causes | Colorectal cancer-specific | Other causes |
| 40-44 | 0.19682 | 0.00295 | 0.19387 |  | 0.08214 | 0.00226 | 0.07988 |
| 45-49 | 0.25645 | 0.0046 | 0.25185 |  | 0.11189 | 0.00338 | 0.10851 |
| 50-54 | 0.59941 | 0.01319 | 0.58622 |  | 0.25835 | 0.0073 | 0.25105 |
| 55-59 | 0.67154 | 0.01663 | 0.65491 |  | 0.27936 | 0.01045 | 0.26891 |
| 60-64 | 1.29656 | 0.03603 | 1.26053 |  | 0.62467 | 0.01998 | 0.60469 |
| 65-69 | 2.12689 | 0.05951 | 2.06738 |  | 1.1235 | 0.03389 | 1.08961 |
| 70-74 | 3.17267 | 0.07824 | 3.09443 |  | 1.87125 | 0.04562 | 1.82563 |


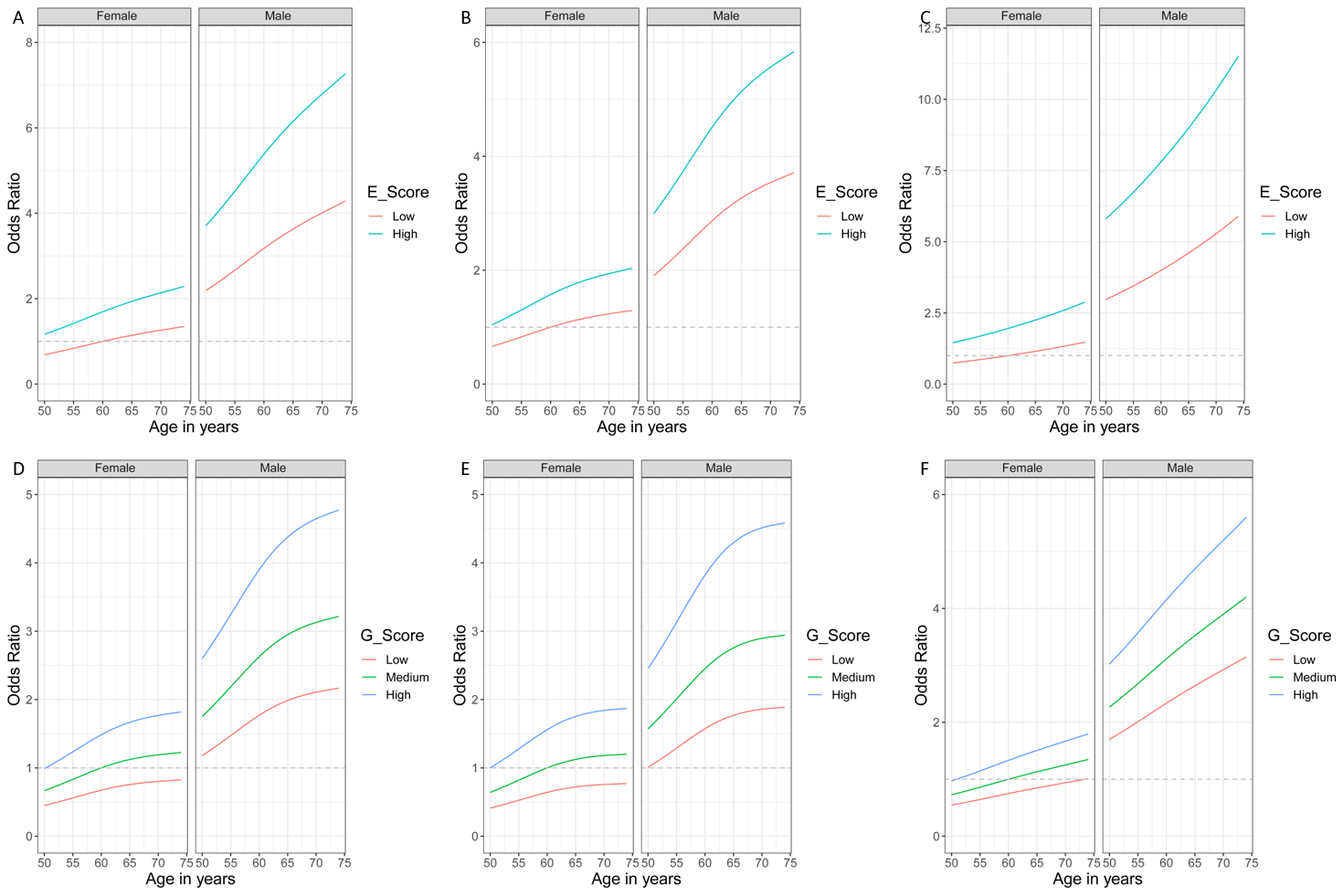


**Figure S1. Odds ratios of E-score (lifestyle score) and G-score (polygenic risk score) according to sex and age.** (A) ORs of lifestyle score for colorectal neoplasm; (B) ORs of lifestyle score for non-advanced adenoma; (C). ORs of lifestyle score for advanced adenoma; (D) ORs of PRS for colorectal neoplasm; (E) ORs of PRS for non-advanced adenoma; (F). ORs of PRS for advanced adenoma.
